# The SARS-CoV-2 ORF6 protein inhibits nuclear export of mRNA and spliceosomal U snRNA

**DOI:** 10.1371/journal.pone.0312098

**Published:** 2024-10-31

**Authors:** Ichiro Taniguchi

**Affiliations:** Institute for Life and Medical Sciences, Kyoto University, Kyoto, Japan; Neyshabur University of Medical Sciences, ISLAMIC REPUBLIC OF IRAN

## Abstract

Severe acute respiratory syndrome coronavirus 2 (SARS-CoV-2) is the causative agent of coronavirus disease 19 (COVID-19). SARS-CoV-2 infection suppresses host innate immunity and impairs cell viability. Among the viral proteins, ORF6 exhibits potent interferon (IFN) antagonistic activity and cellular toxicity. It also interacts with the RNA export factor RAE1, which bridges the nuclear pore complex and nuclear export receptors, suggesting an effect on RNA export. Using the *Xenopus* oocyte microinjection system, I found that ORF6 blocked the export of not only mRNA but also spliceosomal U snRNA. I further demonstrated that ORF6 affects the interaction between RAE1 and nuclear export receptors and inhibits the RNA binding of RAE1. These effects of ORF6 may cumulatively block the export of several classes of RNA. I also found that ORF6 binds RNA and forms oligomers. These findings provide insights into the suppression of innate immune responses and the reduction in cell viability caused by SARS-CoV-2 infection, contributing to the development of antiviral drugs targeting ORF6.

## Introduction

Severe acute respiratory syndrome coronavirus 2 (SARS-CoV-2) is the causative agent of coronavirus disease 19 (COVID-19) [1–3]. COVID-19 is clinically characterized by symptoms such as fever, dry cough, fatigue, and dyspnea. SARS-CoV-2 antagonizes interferon (IFN) production and signaling, thereby suppressing host innate immune responses [4–9]. This IFN antagonism has been implicated in the pathogenesis and severity of COVID-19. Among the viral proteins, ORF6 has been shown to strongly inhibit IFN production and its downstream signaling [4,7,9]. Additionally, the overexpression of ORF6 reduces cell viability [10]. These findings suggest that ORF6 globally impedes cellular gene expression.

The compartmentalization of eukaryotic cells by the nuclear envelope necessitates nuclear RNA export for gene expression [11,12]. Key steps in RNA export include the loading of nuclear export receptors onto RNAs and the docking of the export complex to the nuclear pore complex (NPC), which penetrates the nuclear envelope. The export receptor for bulk mRNAs is the TAP-p15 (also called NXF1-NXT1) heterodimer [13–16], while the receptor for spliceosomal U snRNAs and ribosomal RNAs is CRM1 [17–22]. Some mRNAs, including IFNA1 and cyclin D1 mRNAs, are exported by CRM1 [23–25], although one study has reported that IFNA1 mRNA is exported by TAP-p15 [26]. The export of tRNAs is mediated by Exportin-t or Exportin-5 [27–31]. The RAE1-NUP98 heterodimer, evolutionarily conserved from yeast to frogs to humans, bridges RNA export receptors and the NPC to facilitate RNA export [32–34]. Some pathogenic viruses target the nuclear transport machinery, including RAE1 and NUP98, which impedes host cellular gene expression and/or facilitates viral gene expression [35]. The vesicular stomatitis virus (VSV) matrix (M) protein interacts with RAE1 and NUP98 and blocks RNA export [36–39]. A study on the SARS-CoV-2-human cell protein-protein interactome reported that ORF6 interacts with RAE1 and NUP98 in a similar manner to the VSV M protein [40]. These findings suggest that ORF6 affects mRNA export by targeting RAE1 and NUP98, which is supported by recent studies [6,9,40–45]. However, the effects of ORF6 on the nuclear export of different classes of RNA have not yet been analyzed, and the interaction among full-length purified ORF6, RAE1, and NUP98 proteins remains to be determined.

In the present study using *Xenopus* oocyte microinjection experiments, ORF6 was clearly shown to markedly suppress the nuclear export of not only mRNA but also U snRNA. *In vitro* protein-protein and protein-RNA binding assays using purified recombinant proteins further demonstrated that ORF6 disturbed the interaction between RAE1 and nuclear export receptors and inhibited the RNA binding of RAE1. These effects of ORF6 may globally impede host cell gene expression, including the expression of IFN and IFN-stimulated genes.

## Materials and methods

### DNA constructs

To generate MBP-FLAG-His and MBP-FLAG-RAE1-His plasmids, I initially constructed the pGEX-6p-1-His plasmid in which the 6xHis (His) tag fragment was inserted into the XhoI-NotI sites of pGEX-6p-1. The FLAG tag fragment was then inserted into the BamHI site of pGEX-6p-1-His (pGEX-6p-1-FLAG-His). The GST fragment was removed by PCR from pGEX-6p-1 and the MBP fragment was inserted into the same sites (MBP-FLAG-His). The RAE1 fragment was cloned into the BamHI-XhoI sites of pMBP-FLAG-His (pMBP-FLAG-RAE1-His). To generate the FLAG-RAE1-His and FLAG-NUP98-His plasmids, the FLAG fragment was inserted into the NcoI-BamHI sites of pET-28a (pET-FLAG), and the RAE1 and NUP98 fragments were then cloned into the BamHI-XhoI sites of pET-FLAG (pET-FLAG-RAE1-His and pET-FLAG-NUP98-His). To generate the GST-T7-ORF6-His plasmid, the T7 tag fragment was inserted into the BamHI site and the ORF6 fragment was cloned into the BamHI-XhoI sites of pGEX-6p-1-His (pGEX-T7-ORF6-His). To generate the GST-TAPΔN-His plasmid, the TAP fragment (amino acids 188–619) [46] was cloned into the BamHI-EcoRI sites of pGEX-6p-1-His (pGEX-TAPΔN-His). To generate the p15 plasmid, the p15 fragment was cloned into the NcoI-XhoI sites of pET-28a (pET-p15). To generate the FLAG-TAP plasmid, the FLAG tag fragment was inserted into the HindIII-BamHI sites of pcDNA3 (pcDNA3-FLAG), and the TAP fragment was cloned into the BamHI-EcoRI sites of pcDNA3-FLAG (pcDNA3-FLAG-TAP). The sequences of the primers used in this study are listed in S1 Table.

### Cell culture

HEK293T cells were maintained under an atmosphere containing 5% CO_2_ in Dulbecco’s modified Eagle’s medium (Nacalai Tesque) supplemented with 10% fetal bovine serum (Equitech-Bio, Inc.), 100 units/ml penicillin, and 100 μg/ml streptomycin (Nacalai Tesque).

### Expression and purification of recombinant proteins

Plasmids were transformed into the *Escherichia coli* BL21(DE3) strain and expression was initiated by the addition of 0.2 mM isopropyl β-D-1-thiogalactopyranoside when culture density reached 0.6 (OD_600_). Proteins were then expressed at 20°C overnight. Regarding GST-T7-ORF6-His, cells were harvested and lysed in a French press in lysis buffer (20 mM Tris-HCl, pH 8.0, 0.5 M NaCl, 1 mM 2-mercaptoethanol, 10% glycerol, 10 mM imidazole, 0.1% Nonidet P-40, and a proteinase inhibitor). The supernatant after centrifugation was applied to Ni Sepharose beads (Cytiva). Bound beads were washed five times with Buffer-500 (20 mM Tris-HCl, pH 8.0, 0.5 M NaCl, 1 mM 2-mercaptoethanol, and 10% glycerol) containing 10 mM imidazole, and bound proteins were subsequently eluted in Buffer-500 containing 10–500 mM imidazole. The eluate was dialyzed against Buffer-500 containing 0.1 mM EDTA. Concerning T7-ORF6-His, GST-T7-ORF6-His was treated with PreScission Protease (Cytiva; 2U/μl in Buffer-500) at 4°C overnight. Proteins were applied to glutathione Sepharose beads to remove the GST tag, undigested GST-T7-ORF6-His, and PreScission Protease, and the unbound T7-ORF6-His protein was recovered. MBP-FLAG-His and MBP-FLAG-RAE1-His were purified as GST-T7-ORF6-His. Concerning FLAG-RAE1-His and FLAG-NUP98-His, cells were harvested and lysed in a French press in lysis buffer. The pellet after centrifugation was resuspended in Buffer-500U (20 mM Tris-HCl, pH 8.0, 0.5 M NaCl, 1 mM 2-mercaptoethanol, 6 M urea, and 10 mM imidazole). The supernatant after centrifugation was applied to Ni Sepharose beads. Beads were washed five times with Buffer-500U, and the bound protein was subsequently eluted in Buffer-500U containing 10–500 mM imidazole. The eluate was dialyzed against Buffer-500 containing 0.1 mM EDTA. The GST-TAPΔN:p15 heterodimer was purified as GST-T7-ORF6-His, except that 100 mM NaCl was used instead of 500 mM.

### MBP/GST pull-down

Purified recombinant proteins and whole cell lysates were mixed with RNase A and the indicated recombinant proteins that were pre-bound to Amylose Resin or glutathione Sepharose, equilibrated with RSB100N buffer buffer (10 mM Tris-HCl, pH 7.5, 100 mM NaCl, 2.5 mM MgCl_2_, and 0.1% Nonidet P-40), and rotated at 4°C for 1 h. After washing the beads five times with RSB100N buffer, the bound material was recovered and analyzed by SDS-PAGE and western blotting.

### Western blotting

Primary antibodies were used in TBS-T buffer (20 mM Tris-HCl, pH 7.5, 150 mM NaCl, and 0.1% Tween 20) containing 5% skim milk. Incubations were generally performed at 4°C overnight. As secondary antibodies, HRP-labeled anti-mouse, anti-rabbit, or anti-rat antibodies (Jackson ImmunoResearch) were used in TBS-T buffer containing 5% skim milk. Incubations were conducted as recommended by the manufacturers. The antibodies used in this study are listed in S2 Table.

### *In vitro* transcription

^32^P-labeled RNAs were transcribed in a 10-μl volume containing 20 U T7 RNA Polymerase (Promega), Transcription Buffer (Promega), 1 mM DTT (Promega), 12 U RNasin Plus (Promega), NTP mixture (0.5 mM ATP, CTP, and 0.1 mM UTP, GTP), 1 μg of DNA template, 1 mM m7G(5′)ppp(5′)G RNA Cap Structure Analog (New England Biolabs), and 2.96 TBq/mmol [α-^32^P]UTP (PerkinElmer). Cap Structure Analog was not added for U6Δss snRNA or tRNA^Phe^. After 60 min of incubation at 37°C, RNA was recovered from the supernatants by phenol/chloroform extraction and purified using G-50 micro-columns (Cytiva). RNA was then precipitated with ethanol and dissolved in H_2_O.

### *Xenopus* oocyte microinjection

*Xenopus* oocyte microinjection assays were performed as previously described [47,48]. Briefly, purified recombinant GST or GST-ORF6-His was pre-injected into the cytoplasm of *Xenopus* oocytes. After incubation at 19°C for 12 h, ^32^P-labeled RNAs were injected into the nucleus. After further incubation at 19°C for 0 or 3 h, oocytes were dissected into nuclear and cytoplasmic fractions with a sharp forceps. These fractions were incubated in Homomix buffer (50 mM Tris-HCl, pH 7.5, 5 mM EDTA, 1.5% SDS, 300 mM NaCl, and 1.5 mg/ml proteinase K; Nacalai Tesque) at 50°C for 30 min. RNA was recovered from the supernatants by phenol/chloroform extraction and ethanol precipitation, and then analyzed by denaturing PAGE and autoradiography.

### Transfection

Plasmids were transfected into 70% confluent HEK293T cells in a six-well plate using Lipofectamine 3000 Reagent (Thermo Fisher Scientific), in accordance with the manufacturer’s protocol. After 48 h, the cells were washed twice with phosphate-buffered saline (PBS; 137 mM NaCl, 2.68 mM KCl, 8.1 mM Na_2_HPO_4_, 1.47 mM KH_2_PO_4_) and lysed in RSB100N buffer.

### RNA immunoprecipitation

Recombinant FLAG-RAE1-His protein was pre-bound to Protein A-Sepharose beads via an anti-FLAG antibody. ^32^P-labeled RNAs were mixed with the beads in the absence or presence of purified recombinant T7-ORF6-His protein, and rotated at 20°C for 15 min. After washing the beads five times with RSB100N buffer, the bound material was incubated in Homomix buffer at 50°C for 30 min. RNA was recovered from the supernatants by phenol/chloroform extraction and ethanol precipitation, and then analyzed by denaturing PAGE and autoradiography.

### Electrophoresis mobility shift assay

A ^32^P-labeled RNA probe was mixed with purified recombinant T7-ORF6-His in a 10-μl volume containing 14 mM Tris-HCl (pH 8.0), 150 mM NaCl, 0.7 mM 2-mercaptoethanol, 1.6 mM MgCl_2_, 0.07 mM EDTA, 7% glycerol, and 200 mM sucrose on ice. The mixture was fractionated on a 4% or 6% native polyacrylamide gel in 0.5× TBE at 8.5 V/cm and analyzed by autoradiography. Data were analyzed using GraphPad Prism.

### Statistical analysis

Statistical significance was determined using Student’s *t*-test. Data are presented as the means ± S.D. P-values were calculated by two-tailed paired test.

## Results

### ORF6 directly interacts with RAE1 *in vitro*

**Fig 1:**
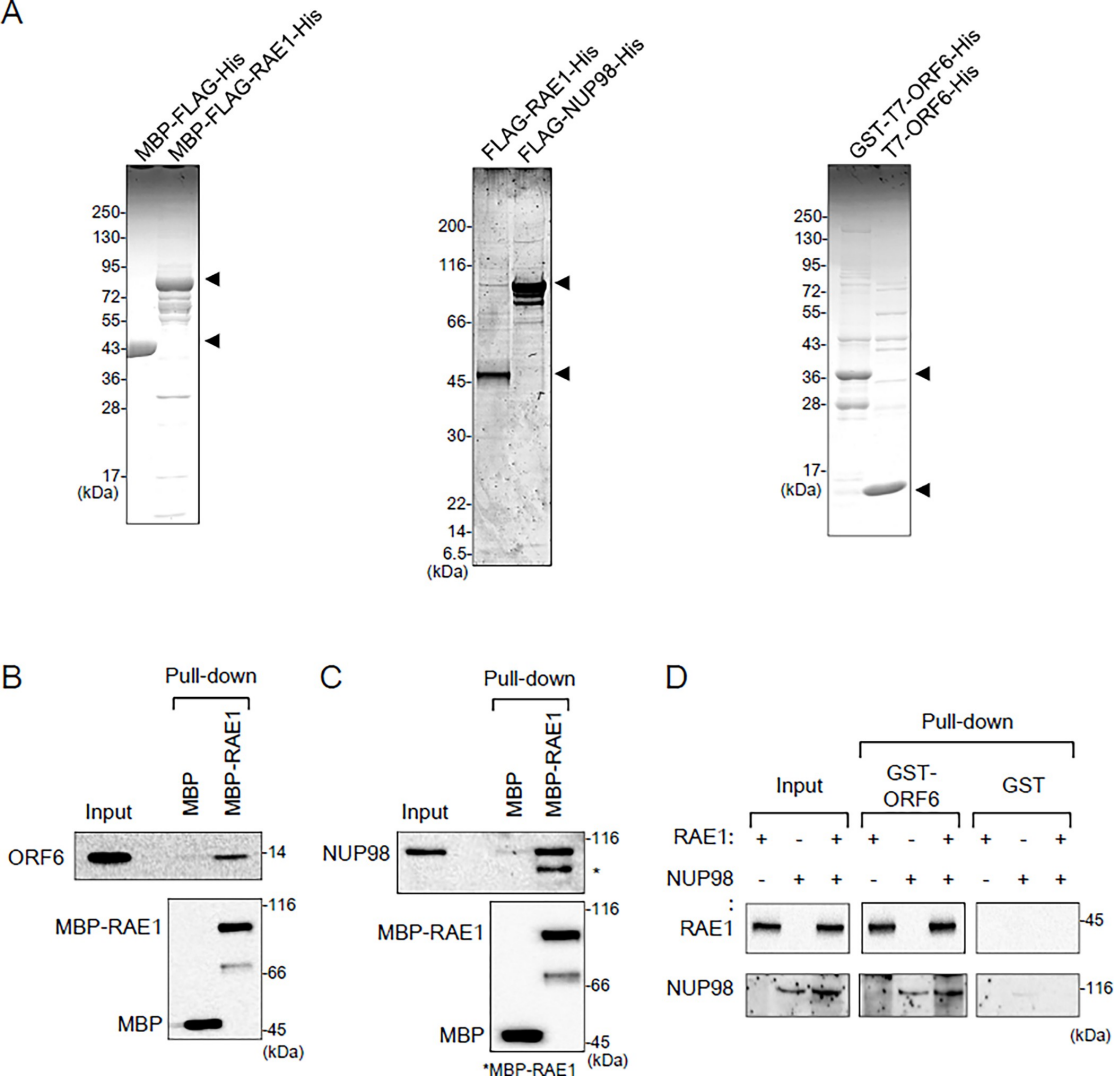
Interaction among full-length ORF6, RAE1, and NUP98 proteins *in vitro*. (A) Coomassie Brilliant Blue (CBB) staining of purified recombinant proteins. (B) A protein mixture containing T7-ORF6-His (ORF6) (1 μg) and RNase A (5 μg) was mixed with MBP-FLAG-His (MBP) or MBP-FLAG-RAE1-His (MBP-RAE1) (1 μg each) that was pre-bound to Amylose Resin. The pulled-down proteins were analyzed by SDS-PAGE and western blotting (WB). (C) A protein mixture containing FLAG-NUP98-His (NUP98) (60 ng) and RNase A (5 μg) was mixed with MBP-FLAG-His (MBP) or MBP-FLAG-RAE1-His (MBP-RAE1) (1 μg each) that was pre-bound to Amylose Resin. Pulled-down proteins were analyzed by SDS-PAGE and western blotting. (D) A protein mixture containing FLAG-RAE1-His (RAE1) (20 ng), FLAG-NUP98-His (NUP98) (60 ng), and RNase A (5 μg) was mixed with GST or GST-T7-ORF6-His (GST-ORF6) (1 μg each) that was pre-bound to glutathione sepharose. Pulled down proteins were analyzed by SDS-PAGE and WB.

To confirm the direct interaction among ORF6, RAE1, and NUP98, I initially performed *in vitro* protein-protein binding assays using purified full-length recombinant proteins (Fig 1A). MBP-RAE1, but not MBP alone, pulled down ORF6 and NUP98 in the presence of RNase A (Fig 1B and 1C). GST-ORF6, but not GST alone, reciprocally pulled down RAE1 in the presence of RNase A (Fig 1D). The interaction between ORF6 and NUP98, if any, appeared to be weak (Fig 1D). These results clearly demonstrate that ORF6 directly interacts with RAE1.

### ORF6 inhibits the nuclear export of RNAs in *Xenopus* oocytes

**Fig 2:**
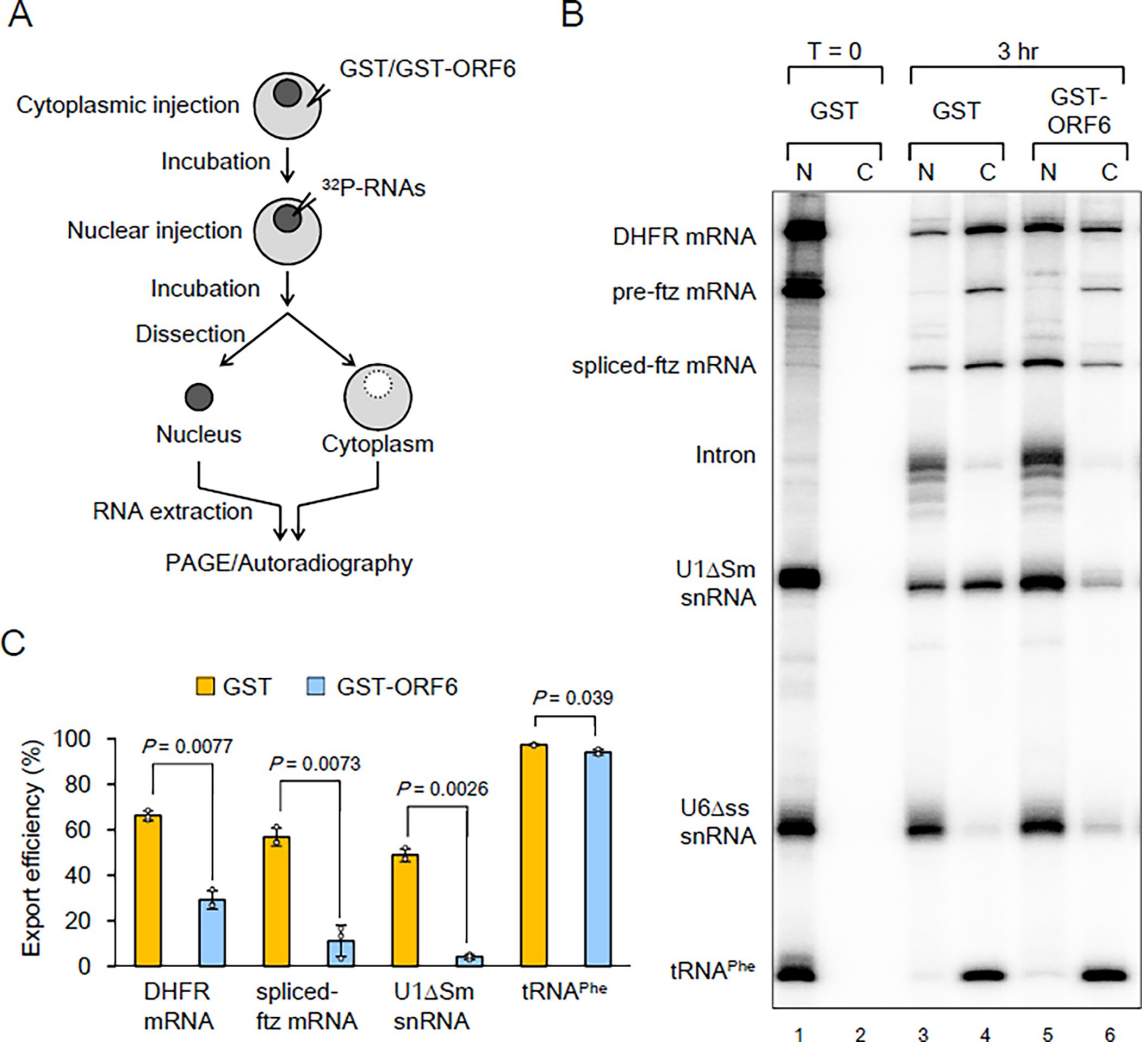
Effects of ORF6 on RNA export in *Xenopus* oocytes. (A) and (B) Purified recombinant GST or GST-T7-ORF6-His (GST-ORF6) (50 fmol/oocyte) was injected into the cytoplasm of *Xenopus* oocytes. After 12 h of incubation, a mixture of *in vitro*-transcribed ^32^P-labeled RNAs containing DHFR mRNA, pre-ftz mRNA, U1ΔSm snRNA, U6Δss snRNA, and tRNA^Phe^ was injected into the nucleus. U6Δss snRNA and tRNA^Phe^ were uncapped, and the other RNAs were m^7^G-capped. RNA was extracted from nuclear (N) and cytoplasmic (C) fractions immediately (0 h; A, lanes 1 and 2) or at 3 h (3 h; A, lanes 3–6) after the injection, and then analyzed by 8% denaturing PAGE and autoradiography. Bands corresponding to the spliced product (spliced-ftz mRNA) and the lariat intron (intron) are indicated. It is likely that the cytoplasmic pre-ftz mRNA band was the result of leakage from tiny holes made by injection needles, rather than export factors. (C) Quantification of the export efficiency of DHFR mRNA, spliced-ftz mRNA, U1ΔSm, and tRNA^Phe^ from three independent experiments performed as in (B) is shown. Values are means (SD).

The direct interaction of ORF6 with RAE1 suggested that ORF6 adversely affected the nuclear export of RNA. To obtain direct evidence of this, I utilized the well-established *Xenopus* oocyte microinjection system to assess the blockade of RNA export by ORF6 (Fig 2A). In this system, RNA export is separated from other steps of gene expression, and its kinetics is evaluated, making it possible to clearly demonstrate ORF6’s effects on the export of different classes of RNA. To achieve this, a mixture of ^32^P-labeled RNAs containing intronless DHFR mRNA, an intron-containing ftz mRNA precursor (pre-ftz mRNA), U1ΔSm snRNA, U6Δss snRNA, and tRNA^Phe^ was microinjected into the nuclei of *Xenopus* oocytes that were pre-injected with purified recombinant GST. U1ΔSm snRNA is exported from the nucleus but, unlike wild-type U1 snRNA, is not re-imported to the nucleus due to the mutation of the Sm site [49]. U6Δss snRNA is neither imported nor exported from the nucleus, as the single-stranded region is crucial for U6 snRNA import [50]. Immediately thereafter, all RNAs localized in the nucleus (Fig 2B, lanes 1 and 2). After 3 h of incubation, pre-ftz mRNA was efficiently spliced, and a proportion of spliced-ftz mRNA was exported to the cytoplasm, while the excised intron remained in the nucleus (Fig 2B, lanes 3 and 4). Intronless DHFR mRNA, U1ΔSm snRNA, and tRNA^Phe^ were also partially or completely exported, whereas U6Δss snRNA control remained in the nucleus (Fig 2B, lanes 3 and 4).

To investigate whether ORF6 blocks RNA export, purified recombinant GST-ORF6 was injected into the cytoplasm prior to the nuclear injection of RNAs. The pre-injection of ORF6 strongly inhibited the export of DHFR mRNA, spliced-ftz mRNA, and U1ΔSm snRNA (Fig 2B, lanes 5 and 6, and 2C for quantification). These results clearly indicated that ORF6 blocked TAP-dependent mRNA and CRM1-dependent U snRNA export. The inhibition of tRNA export was negligible, likely due to tRNA being completely exported during the 3 h of incubation. An analysis of nuclear export 30 min after the RNA injection revealed a more prominent inhibition of tRNA export (S1 Fig). The splicing reaction of pre-ftz mRNA was not inhibited.

### ORF6 affects the interaction of RAE1 with RNA export receptors *in vitro*

**Fig 3:**
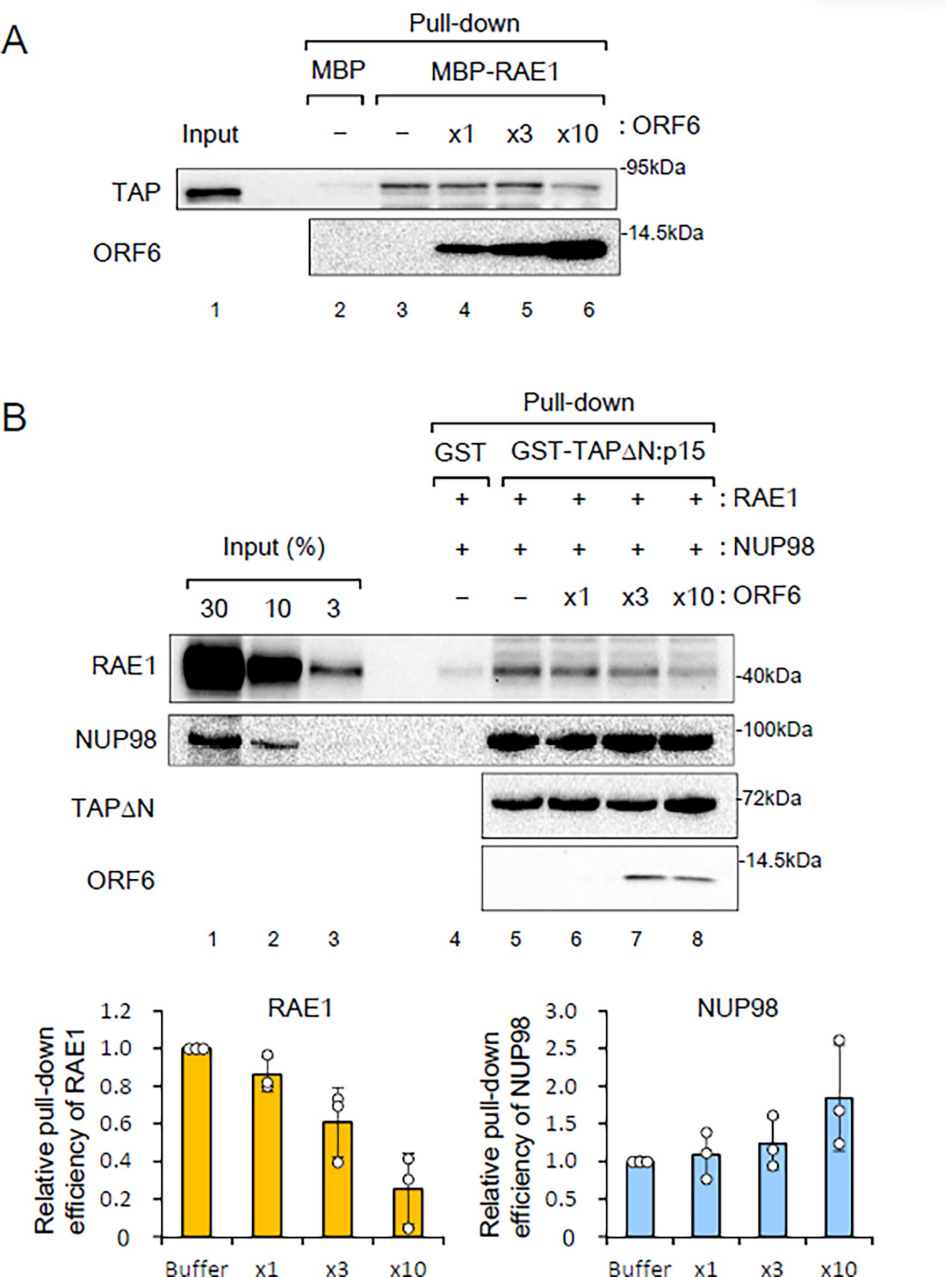
Effects of ORF6 on protein interactions of RAE1 *in vitro*. (A) pcDNA3-FLAG-TAP was transfected into HEK293T cells. Whole-cell lysates containing T7-ORF6-His (0, 1, 3, and 10 μg; -, x1, x3, and x10, respectively) and RNase A (25 μg) were mixed with MBP-FLAG-His (MBP) or MBP-FLAG-RAE1-His (MBP-RAE1) (2 μg each) that was pre-bound to Amylose Resin. Pulled-down proteins were analyzed by SDS-PAGE and WB. (B) A protein mixture containing FLAG-RAE1-His (RAE1) (1 μg), FLAG-NUP98-His (NUP98) (0.5 μg), T7-ORF6-His (ORF6) (0, 2.5, 7.5, and 25 μg; -, x1, x3, and x10, respectively), and RNase A (50 μg) was mixed with GST (2.5 μg) or the GST-TAPΔN:p15 complex (2.5 μg TAPΔN) that was pre-bound to glutathione Sepharose. Pulled-down proteins were analyzed by SDS-PAGE and WB. Quantification of the relative pull-down efficiency from three independent experiments is shown. The efficiency of the buffer is set to 1. Values are means (SD).

The RAE1-NUP98 heterodimer is involved in RNA export by bridging NPC and RNA export receptors [32–34]. To elucidate the mechanisms by which ORF6 inhibits RNA export, I examined its effects on the interactions of RAE1 with the mRNA export receptor TAP-p15. A pull-down assay was conducted using purified MBP-RAE1 and human embryonic kidney (HEK) 293T cell lysates. TAP was successfully pulled down by MBP-RAE1, but not by MBP alone (Fig 3A, lanes 2 and 3), indicating that TAP interacted with RAE1 in the presence of cell lysates. Notably, the interaction between RAE1 and TAP was inhibited by the addition of ORF6 (Fig 3A, lane 6). These results suggest that ORF6 disrupts the interaction between the RAE1-NUP98 heterodimer and RNA export receptors.

To further investigate the role of ORF6, I performed a GST pull-down experiment using purified recombinant proteins, including TAP, p15, RAE1, NUP98, and ORF6, in the absence of cell lysates. The N-terminal deletion mutant was utilized in this experiment, as it exhibits a higher degree of purity than the full-length protein [46]. GST-TAPΔN:p15, but not GST alone, successfully pulled down RAE1 and NUP98 (Fig 3B, lanes 4 and 5), suggesting that TAP-p15 and RAE1-NUP98 form a tetrameric complex. The interaction of TAP-p15 with RAE1, but not with NUP98, was inhibited in a dose-dependent manner by the addition of ORF6 (Fig 3B, lanes 6–8, and quantification graphs), indicating that ORF6 inhibits the interaction between RAE1 and TAP-p15.

### ORF6 inhibits the RNA binding of RAE1 *in vitro*

**Fig 4:**
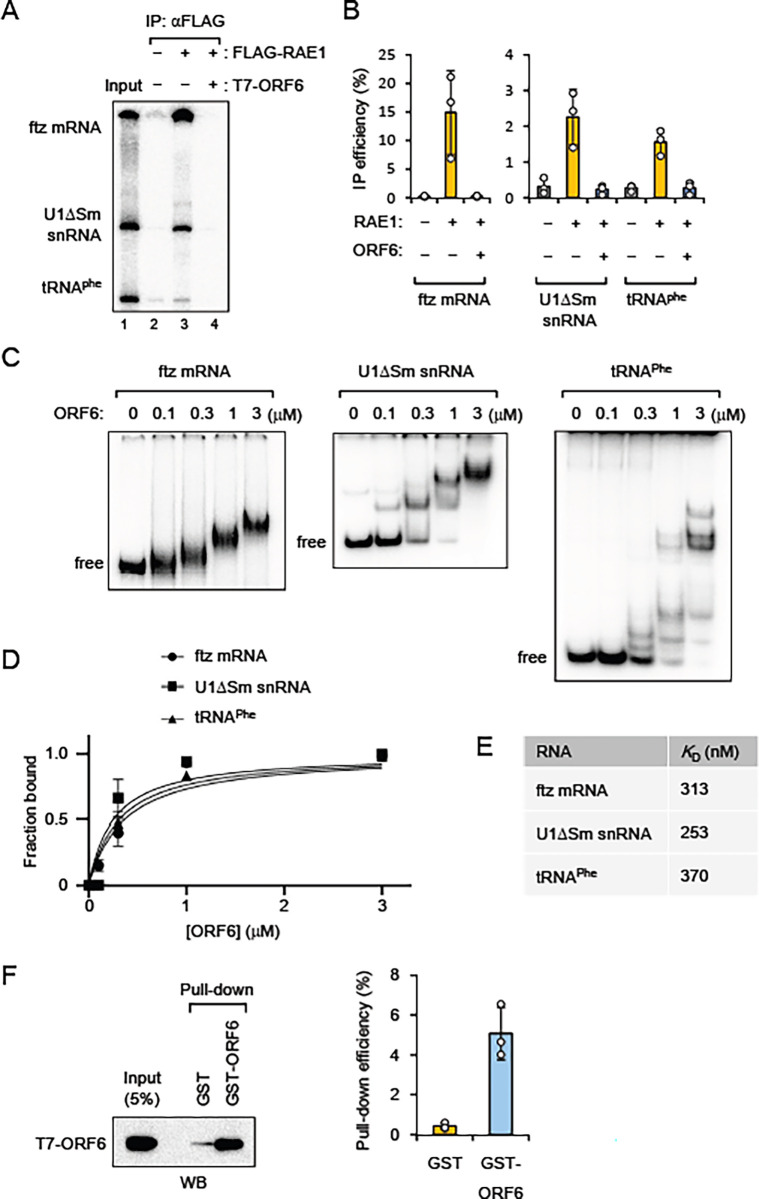
Effects of ORF6 on the RNA binding of RAE1 *in vitro*. (A) A mixture of *in vitro*-transcribed ^32^P-labeled RNAs containing intronless ftz mRNA, U1ΔSm snRNA, and tRNA^Phe^ was incubated with or without purified FLAG-RAE1-His (FLAG-RAE1) (2 μg) that was pre-bound to Protein A Sepharose via an anti-FLAG antibody in the absence or presence of T7-ORF6-His (T7-ORF6) (4 μg). Co-immunoprecipitated RNAs were analyzed by 8% denaturing PAGE and autoradiography. (B) Quantification of the relative pull-down efficiency from three independent experiments performed as in (A) is shown. Values are means (SD). (C) ^32^P-labeled RNA was incubated in the absence or presence of T7-ORF6-His (ORF6) (0.1, 0.3, 1 and 3 μM) on ice. The sample was analyzed by native PAGE and autoradiography. (D) Quantification from three independent experiments performed as in (C) is shown. Values are means (SD). (E) The equilibrium dissociation constant (*K*_D_) was calculated from the quantification in (D). (F) A protein mixture containing T7-ORF6-His (ORF6) (1 μg) and RNase A (5 μg) was pulled down by GST or GST-T7-ORF6-His (GST-ORF6) (1 μg each). Pulled-down proteins were separated by SDS-PAGE and detected by WB. Quantification of the pull-down efficiency of T7-ORF6-His from three independent experiments is shown. Values are means (SD).

Given that a previous study reported that the RNA-binding activity of RAE1 contributes to RNA export [51], I investigated the effects of ORF6 on the RNA binding of RAE1. Although studies using electrophoresis mobility shift assays reported that the ORF6-derived peptide inhibits the RNA binding of RAE1-NUP98 fragments [44,45], I could not detect the shifted band using full-length proteins. Therefore, I performed an RNA immunoprecipitation assay. A mixture of ^32^P-labeled RNAs containing intronless ftz mRNA, U1ΔSm snRNA, and tRNA^Phe^ was incubated with purified recombinant FLAG-RAE1. All RNAs were successfully immunoprecipitated with an anti-FLAG antibody, indicating that RAE1 bound to the RNAs (Fig 4A, lane 3). However, when purified recombinant ORF6 was added, RNAs were not precipitated (Fig 4A, lane 4, and 4B for quantification), suggesting that ORF6 inhibits the RNA binding of RAE1.

I performed an ultraviolet (UV) cross-linking assay to confirm the inhibitory effect of ORF6 on the RNA binding of RAE1. In this assay, purified recombinant RAE1 protein was incubated with ^32^P-labeled RNA and then irradiated with UV light. If RAE1 binds to RNA, it is cross-linked to the RNA and labeled with ^32^P. After incubation with RNase A to digest the RNA moiety, the sample was separated by SDS-PAGE and detected by autoradiography. Although a clear band corresponding to RAE1 was not detected, a band corresponding to approximately 10 kDa was observed (S2 Fig), suggesting that ORF6 exhibited RNA-binding activity.

To confirm the RNA-binding activity of ORF6, an electrophoresis mobility shift assay was performed (Fig 4C–4E). As expected, ORF6 exhibited RNA-binding activity, with slower migrating complexes detected in a dose-dependent manner. This suggests that ORF6 proteins may interact with one other on a single RNA molecule. To investigate whether ORF6 proteins interacted with each other, a GST pull-down assay was conducted. GST-ORF6, but not GST alone, successfully pulled down T7-ORF6 in the presence of RNase A (Fig 4F). These results indicate that ORF6 can form oligomers.

## Discussion

The present results clearly demonstrated that ORF6 inhibited both TAP-dependent and CRM1-dependent RNA export pathways in *Xenopus* oocytes (Fig 2). Regarding the underlying molecular mechanisms, I showed that ORF6 inhibited the interaction between RAE1 and TAP-p15 (Fig 3) and diminished the RNA binding of RAE1 (Fig 4) [44,45]. Furthermore, I found that ORF6 proteins exhibited RNA-binding and dimer formation activities (Fig 4) [52]. This study revealed the inhibitory effects of ORF6 on RNA export, which appear to promote virus-specific gene expression while blocking cellular gene expression, including that of genes involved in cell viability and innate immunity [4,7,9,10].

A model for the role of the SARS-CoV-2 ORF6 protein in the reduction of cell viability and suppression of host innate immunity is shown in S3 Fig. ORF6 directly interacts with RAE1, thereby inhibiting the interactions between RAE1 and nuclear export receptors and diminishing the RNA binding of RAE1. ORF6 also suppresses the cellular localization of both RAE1 and NUP98 [43]. These non-mutually exclusive, multilayered effects of ORF6 have the potential to cumulatively inhibit the nuclear export of various classes of RNA and block global cellular gene expression, including that of IFN and IFN-stimulated genes. Therefore, SARS-CoV-2 reduces cell viability and suppresses host innate immunity.

Some pathogenic viruses often impede various steps of cellular gene expression, including transcription, splicing, RNA export, and translation, thereby facilitating viral gene expression [35,53,54]. Because SARS-CoV-2 replicates in the cytoplasm, viral gene expression is essentially unaffected by the inhibition of transcription, splicing, and RNA export. Therefore, the complete suppression of RNA export is an effective strategy for SARS-CoV-2. The development of agents that target the inhibition of RNA nuclear export by viruses presents an important challenge to restore host gene expression mechanisms and induce innate immune responses.

The *Xenopus* oocyte microinjection system, which separates RNA export from transcription, clearly and quantitatively demonstrated that ORF6 strongly inhibited not only the TAP-dependent export pathway but also the CRM1-dependent pathway. These results suggest that ORF6 inhibited the CRM1-dependent export of IFNA1 and cyclin D1 mRNAs, as well as bulk mRNA and U snRNA [23–25]. CRM1 also exports transcription factors and cell cycle regulators [55]. Previous studies have demonstrated that ORF6 inhibited the nuclear localization of STAT1 and IRF5 proteins [4,6–9]. Although the transport of some RNAs and proteins was affected, ORF6 weakly inhibited Xpo-t- and Xpo-5-dependent tRNA export. Further studies are warranted to clarify whether RAE1 engages in specific transport pathways. Examining ORF6 should provide more detailed insights into nucleocytoplasmic transport.

## Supporting information

S1 Table Primers used in this study. (XLSX)

S2 Table Antibodies used in this study. (XLSX)

S1 Fig Effects of ORF6 on RNA export in *Xenopus* oocytes. (A) Purified recombinant GST or GST-ORF6-His (50 fmol/oocyte) was pre-injected into the cytoplasm of *Xenopus* oocytes. After a 12-hour incubation, a mixture of *in vitro*-transcribed ^32^P-labeled RNAs containing DHFR mRNA, pre-ftz mRNA, U6Dss snRNA, and tRNA^Phe^ was injected into the nucleus. RNA was immediately extracted from nuclear (N) and cytoplasmic (C) fractions (T = 0) or 30 min after the injection, and then analyzed by 8% denaturing PAGE and autoradiography. Bands corresponding to the spliced product (spliced-ftz mRNA) and the lariat intron (intron) are indicated. (B) Quantification of the export of tRNA^Phe^. Values are the means (SD) (n = 3).
(PDF)

S2 Fig RNA binding of ORF6 *in vitro*. ^32^P-labeled U1 snRNA was incubated with T7-ORF6-His (1 μg) at 30°C for 20 min. After the incubation, the sample was irradiated with UV light (200 mJ/cm^2^), and treated with RNase A. The sample was immunoprecipitated using an antibody against the T7 tag or the Myc tag. The immunoprecipitated protein was analyzed by SDS-PAGE and autoradiography.
(PDF)

S3 Fig A model of the mechanism by which the SARS-CoV-2 ORF6 protein inhibits RNA export. See the Discussion section for details.
(PDF)

S1 Raw images Original gel/blot images. (PDF)
