# Supplementary figures and images for: The SARS-CoV-2 ORF6 protein inhibits nuclear export of mRNA and spliceosomal U snRNA
Source: PLoS One. 2024 Oct 31;19(10):e0312098. doi: 10.1371/journal.pone.0312098 (PMC11527279; doi:10.1371/journal.pone.0312098)

**A**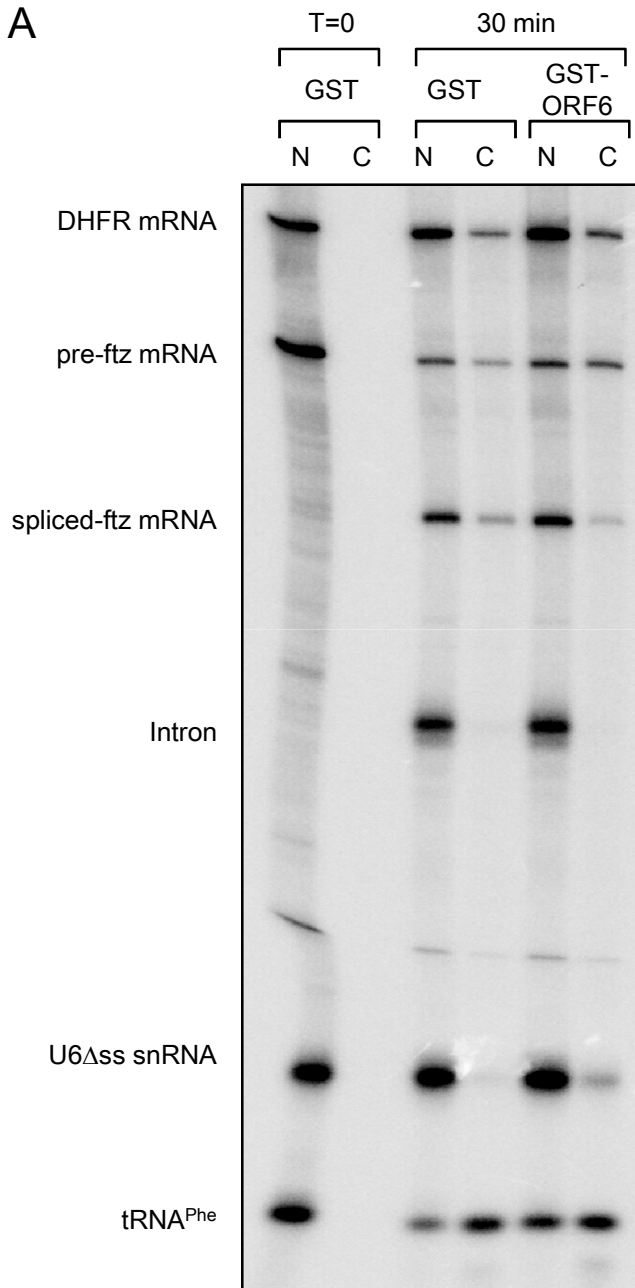**B**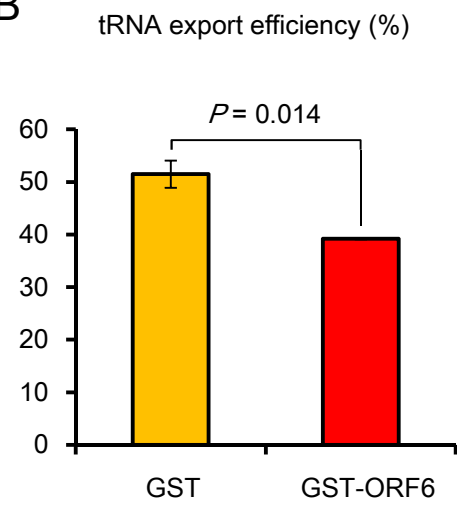

Supplement: S1 Fig — (A) Purified recombinant GST or GST-ORF6-His (50 fmol/oocyte) was pre-injected into the cytoplasm of Xenopus oocytes. After a 12-hour incubation, a mixture of in vitro-transcribed 32P-labeled RNAs containing DHFR mRNA, pre-ftz mRNA, U6Dss snRNA, and tRNAPhe was injected into the nucleus. RNA was immediately extracted from nuclear (N) and cytoplasmic (C) fractions (T = 0) or 30 min after the injection, and then analyzed by 8% denaturing PAGE and autoradiography. Bands corresponding to the spliced product (spliced-ftz mRNA) and the lariat intron (intron) are indicated. (B) Quantification of the export of tRNAPhe. Values are the means (SD) (n = 3). (PDF) [file pone.0312098.s003.pdf]

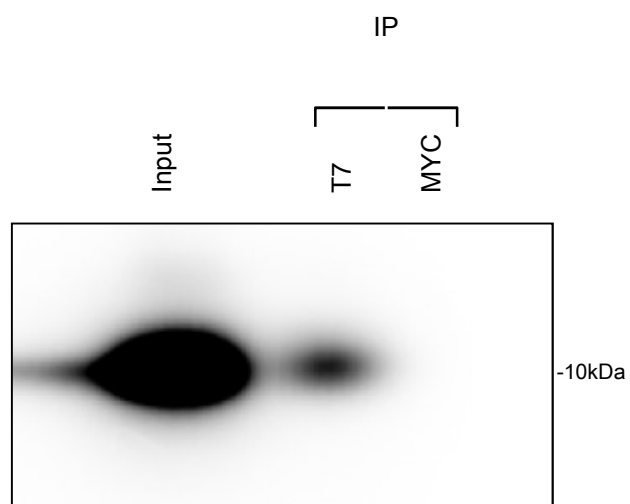

Supplement: S2 Fig — 32P-labeled U1 snRNA was incubated with T7-ORF6-His (1 μg) at 30°C for 20 min. After the incubation, the sample was irradiated with UV light (200 mJ/cm2), and treated with RNase A. The sample was immunoprecipitated using an antibody against the T7 tag or the Myc tag. The immunoprecipitated protein was analyzed by SDS-PAGE and autoradiography. (PDF) [file pone.0312098.s004.pdf]

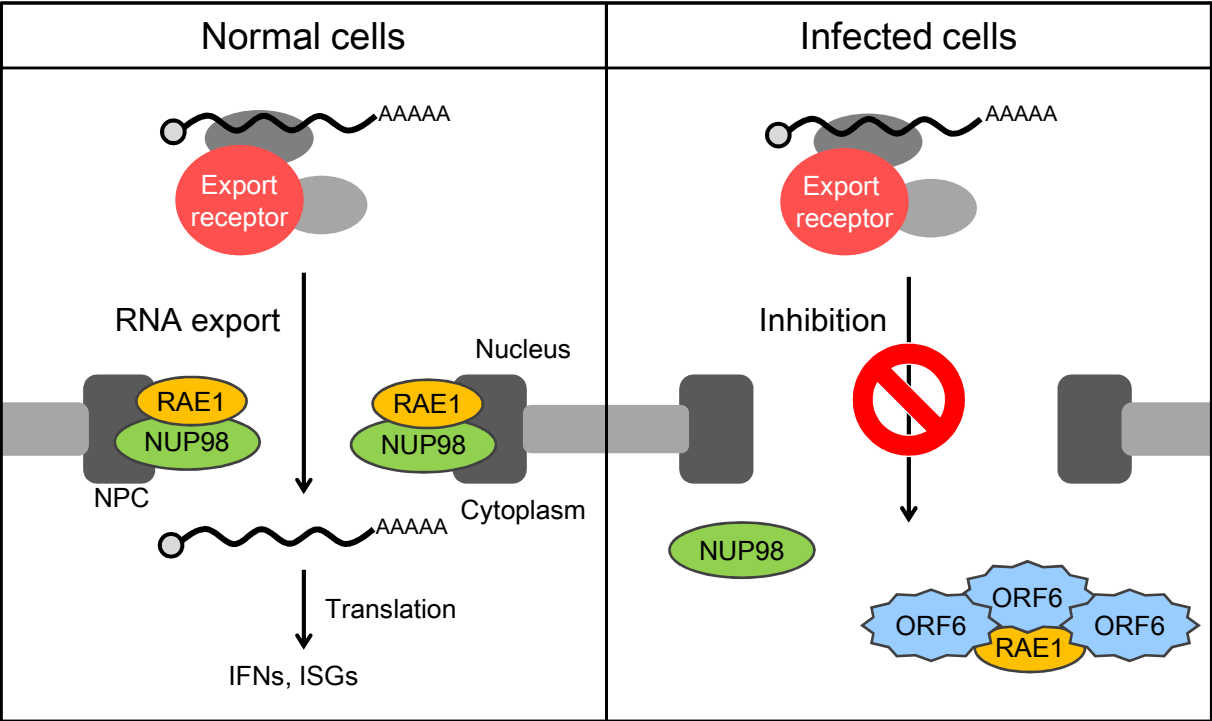

Supplement: S3 Fig — See the Discussion section for details. (PDF) [file pone.0312098.s005.pdf]
