# Supplementary material for: The SARS-CoV-2 ORF6 protein inhibits nuclear export of mRNA and spliceosomal U snRNA
Source: PLoS One. 2024 Oct 31;19(10):e0312098. doi: 10.1371/journal.pone.0312098 (PMC11527279; doi:10.1371/journal.pone.0312098)

Fig 1A

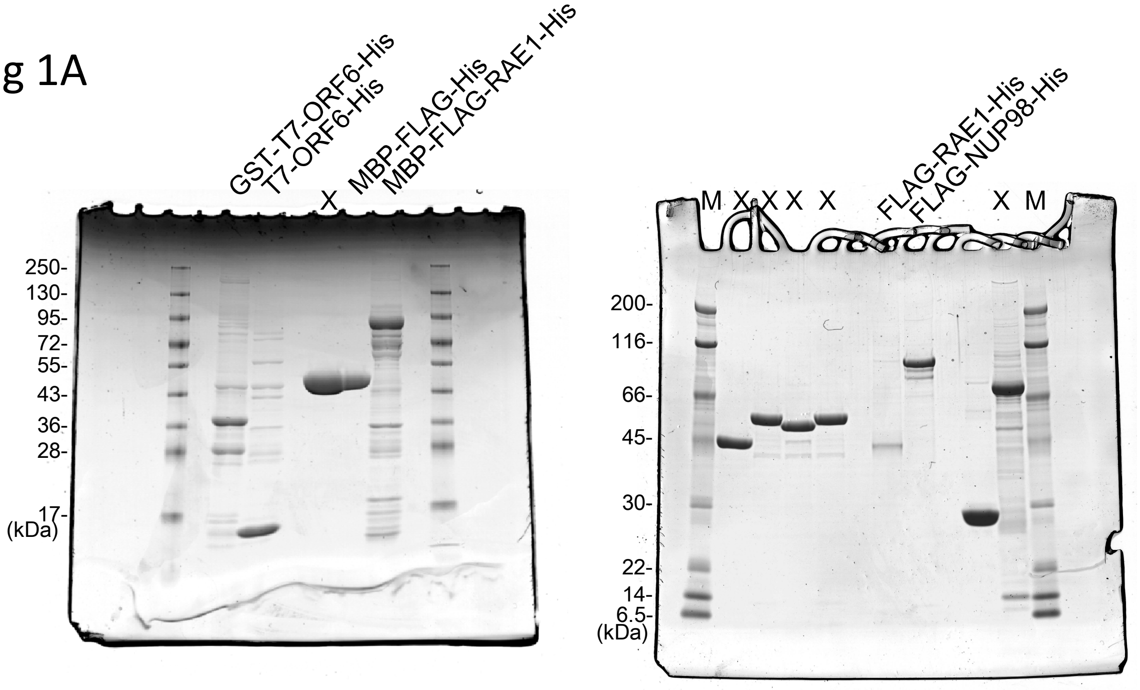

Fig 1B

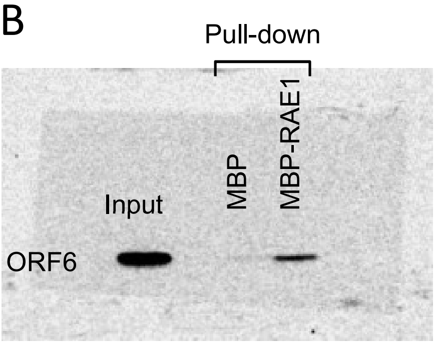

Fig 1C

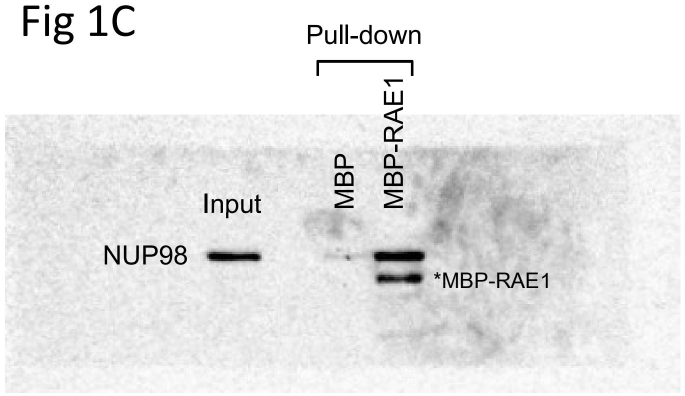

Fig 1B

Fig 1C

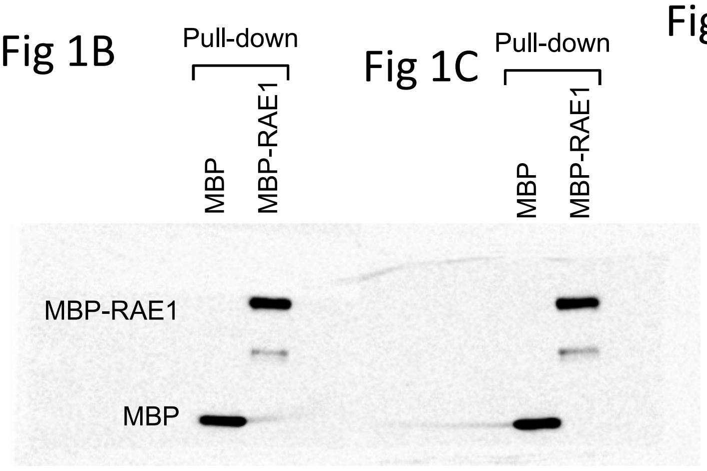

Fig 1D

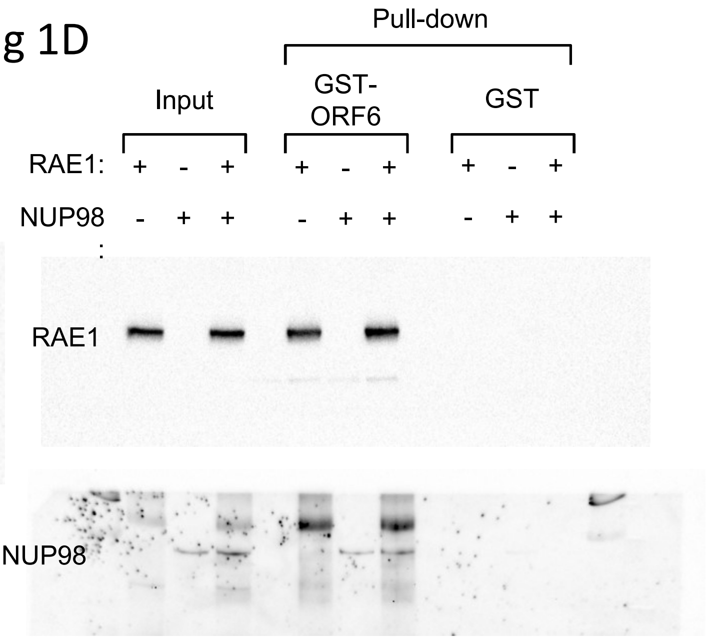

Fig 2B

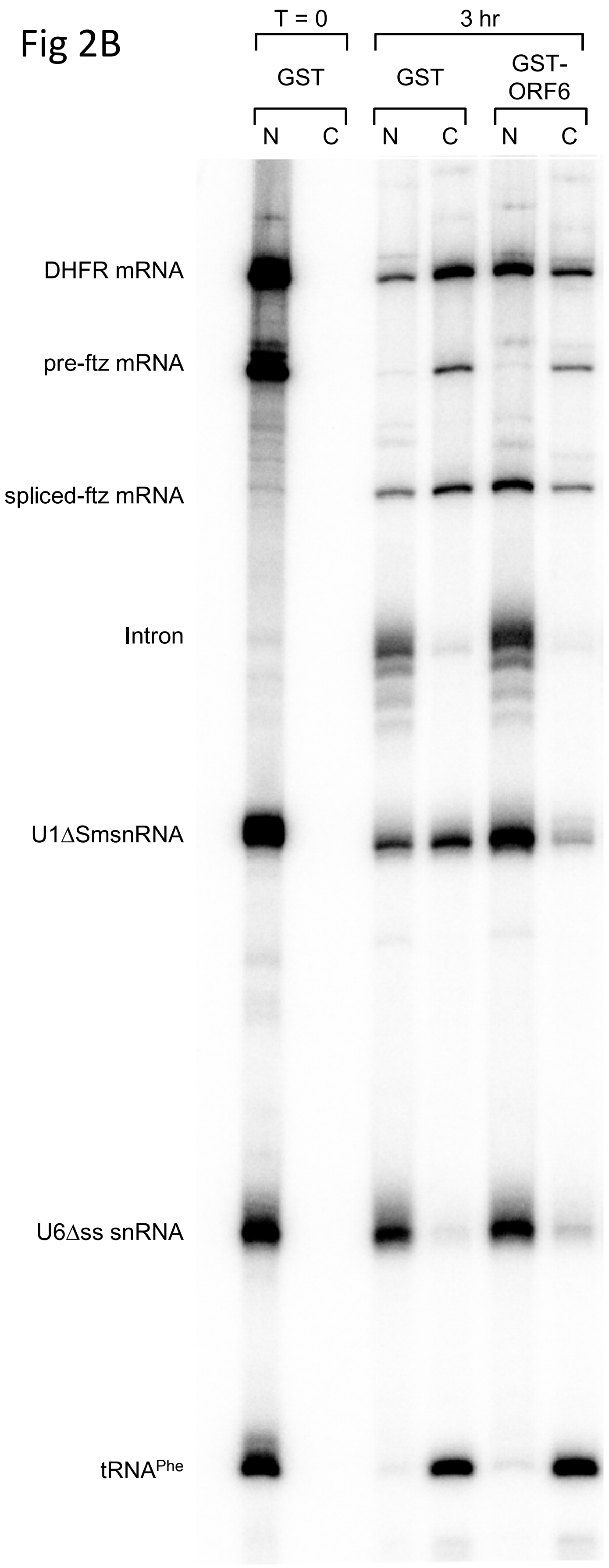
T = 0
3 hr
GST
GST
GST-
ORF6
N
C
N
C
N
C
DHFR mRNA
pre-ftz mRNA
spliced-ftz mRNA
Intron
U1ΔSmsnRNA
U6Δss snRNA
tRNAPhe

Fig 3A

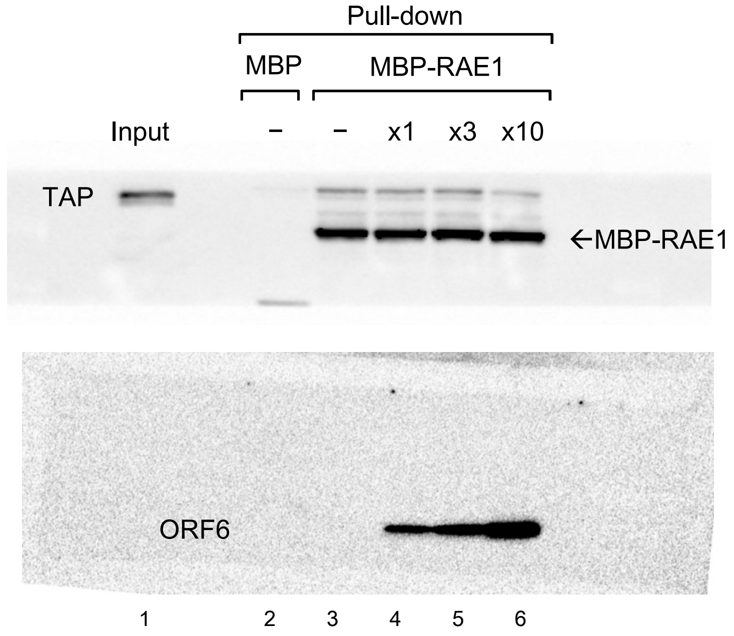
Pull-down
MBP
MBP-RAE1
Input
−
−
x1
x3
x10
TAP
←MBP-RAE1
ORF6
1
2
3
4
5
6

Fig 3B

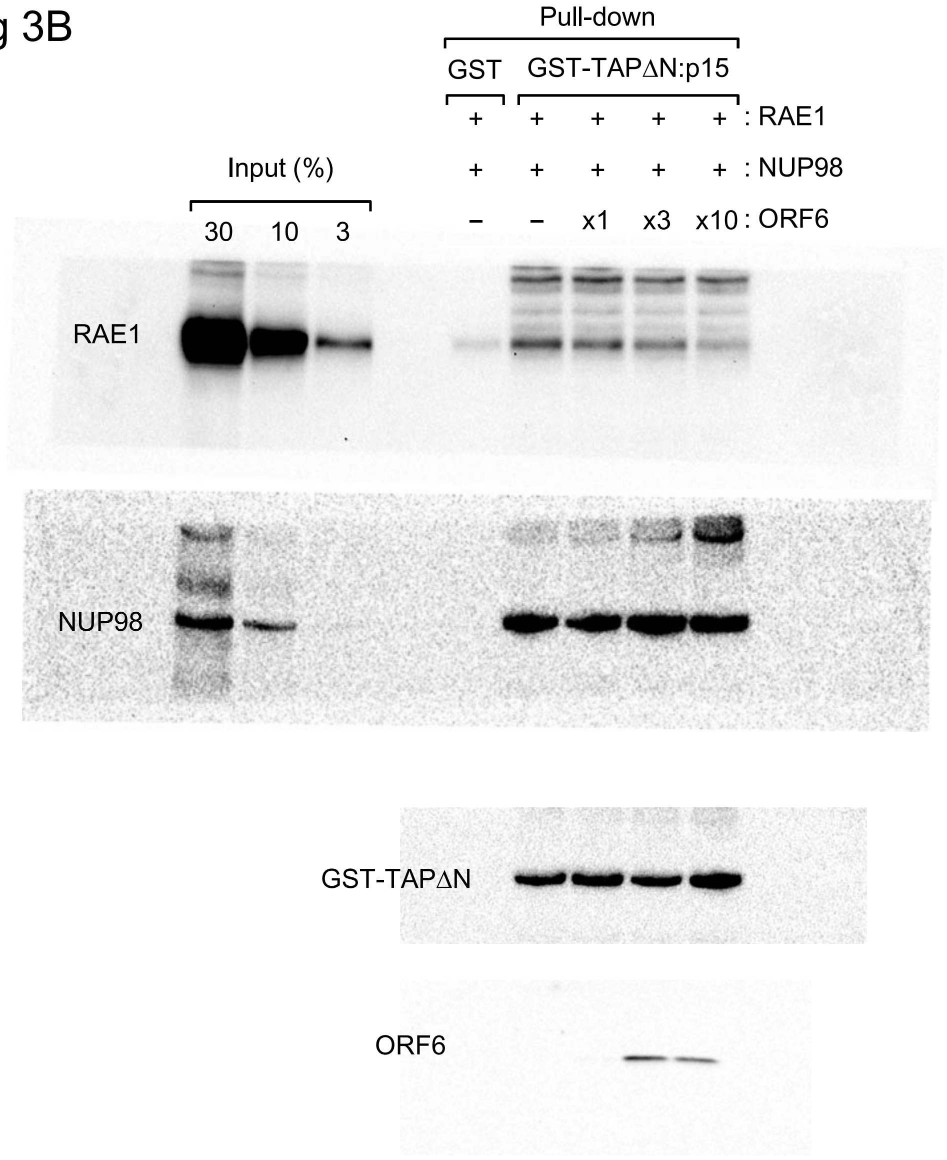
Pull-down
GST
GST-TAPΔN:p15
+ + + + + : RAE1
+ + + + + : NUP98
− − x1 x3 x10 : ORF6
Input (%)
30
10
3
RAE1
NUP98
GST-TAPΔN
ORF6

Fig 4A

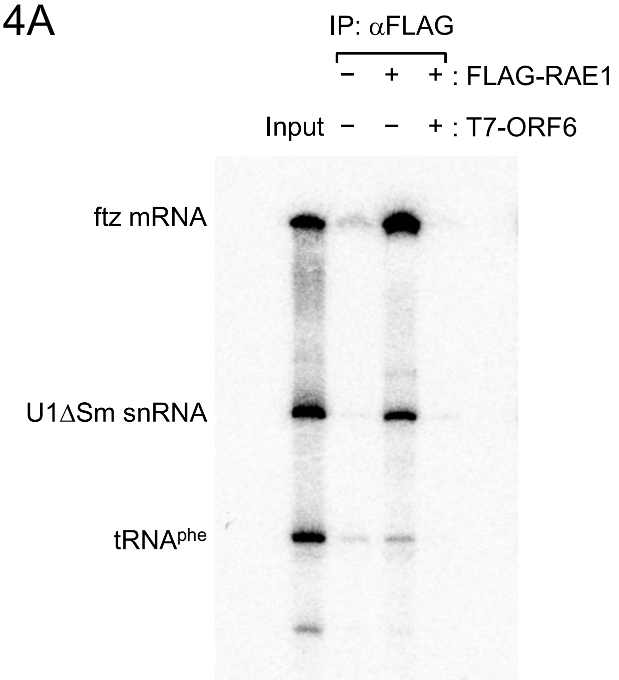

Fig 4C

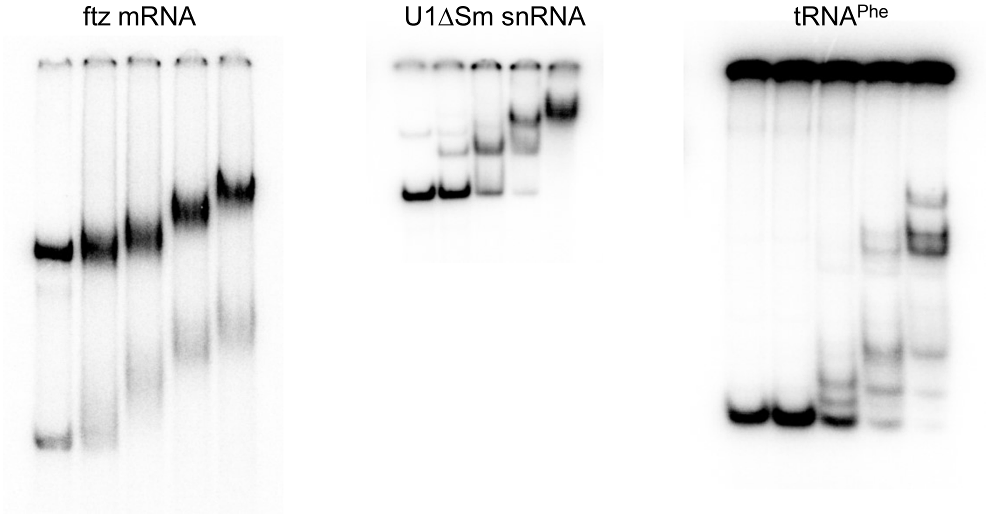

Fig 4F

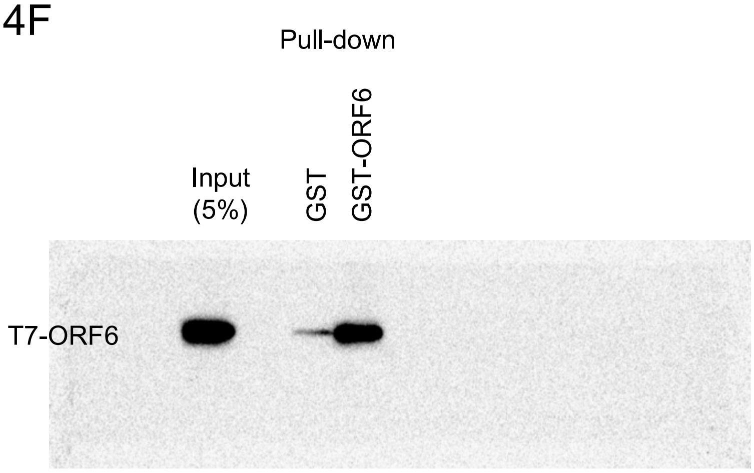

Supplement: S1 Raw images — (PDF) [file pone.0312098.s006.pdf]
